# Bilateral choroidal tuberculoma in a patient of miliary tuberculosis

**DOI:** 10.1186/s12348-025-00509-2

**Published:** 2025-10-24

**Authors:** Tanya Jain, Sachit Mahajan, Aishwaraya Kanagaraj, Vivek Jha

**Affiliations:** 1 Vitreo-retina and Uvea Department, Dr Shroff’s Charity Eye Hospital, 5072, Kedarnath Road, Daryaganj, New Delhi, 110002 India; 2 Dr Shroff’s Charity Eye Hospital, 5072, Kedarnath Road, Daryaganj, New Delhi, 110002 India

**Keywords:** Anti-VEGF, Choroidal TB granuloma, Miliary TB

## Abstract

**Background:**

Choroidal granuloma is one of the common manifestations of ocular tuberculosis. Tubercles indicate hematogenous dissemination of the disease. Tubercular granulomas respond to Anti-Tubercular Treatment (ATT) and systemic corticosteroids. However, in some patients with large granulomas involving macula, adjunct treatment with intravitreal anti-VEGF may be required for prompt management of the granuloma.

**Findings:**

We report a case of bilateral Tubercular granuloma in a patient with miliary Tuberculosis (TB). The patient was an immunocompetent young female with miliary tuberculosis. Mantoux was positive. HRCT chest suggested miliary TB. The patient was already on ATT. Clinical examination showed multiple TB granulomas in both eyes, with a large granuloma involving the fovea in the left eye. She underwent intravitreal injection of the anti-VEGF drug bevacizumab (1.25 mg/0.05 mL) (off-label use) with moxifloxacin (500 µg/0.1 mL) (off-label use) in the left eye. She was continued on ATT and was started on oral steroids. After seven weekly intravitreal injections in the left eye at the second-month follow-up, lesions were consolidated and scarred. Optical coherence tomography showed a decrease in the size of the choroidal bump.

**Conclusion:**

Weekly administration of intravitreal Anti-Vascular endothelial growth factor(VEGF) and moxifloxacin, along with ATT and oral corticosteroids, has controlled inflammation and has caused consolidation and scarring of TB granulomas in a patient with miliary TB.

## Background

Tuberculosis is caused by Mycobacterium tuberculosis. It is well known to involve most ocular tissue, including the orbit and, most commonly, the uveal tissue, due to its high vascularity, which allows easy hematogenous spread of infection [1]. The innate immunity limits the infection through a robust adaptive immune response by forming epithelioid cell granuloma with central areas of caseation necrosis [2, 3]. Tuberculosis-related granulomas usually respond well to a combination of systemic steroids and specific antitubercular therapy (ATT) [2]. This case demonstrates a patient of miliary tuberculosis with bilateral tuberculous granulomas, managed with intravitreal moxifloxacin and intravitreal anti-VEGF, along with ATT and systemic steroids.

## Case presentation

**Fig. 1:**
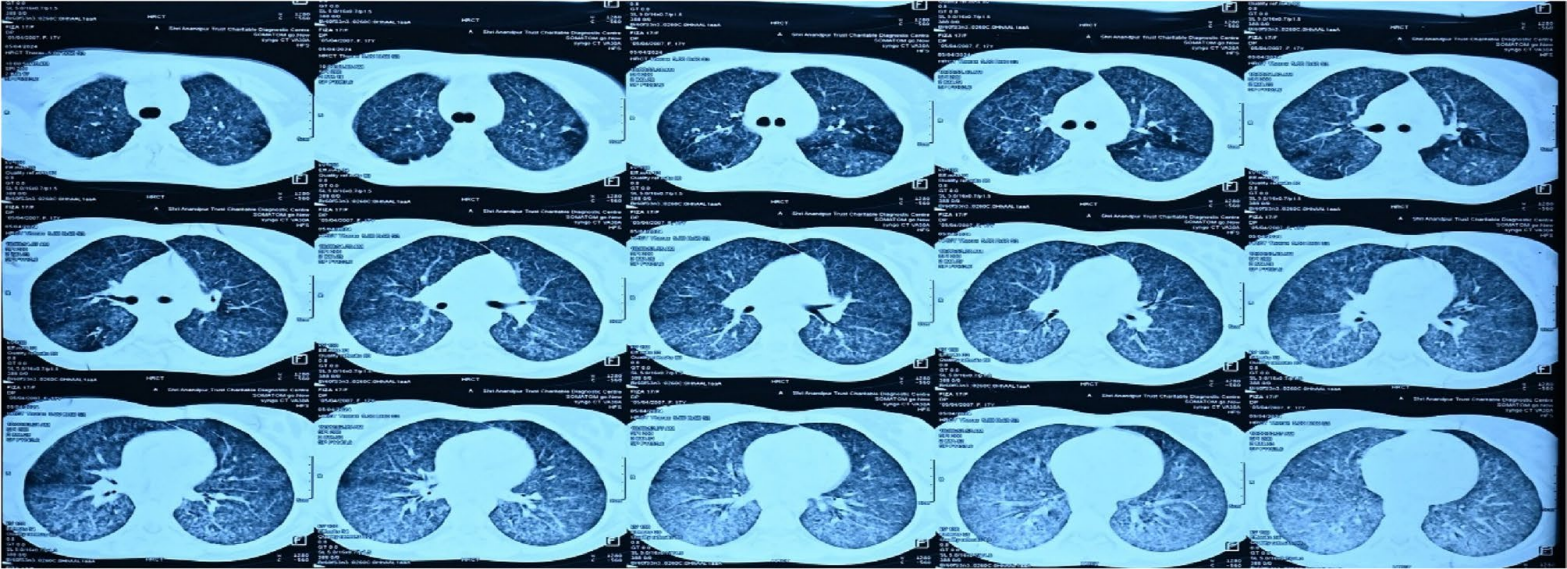
HRCT chest showed bilateral diffuse multiple tiny nodular lesions in the lung parenchyma, suggestive of miliary TB

A 21-year-old female presented with a history of diminished vision in both eyes (BE) for the past month. There was a history of shortness of breath and headache 2 months earlier. Her Mantoux test was positive and showed 12*12 mm induration. TB gold test was positive, and the high-resolution computed tomography (HRCT) of the chest revealed the presence of multiple tiny nodular lesions in bilateral lung fields with no evidence of cavitation, suggestive of miliary tuberculosis (Fig. 1). She was diagnosed with miliary tuberculosis and was started on ATT by the physician 1 month back.

**Fig. 2:**
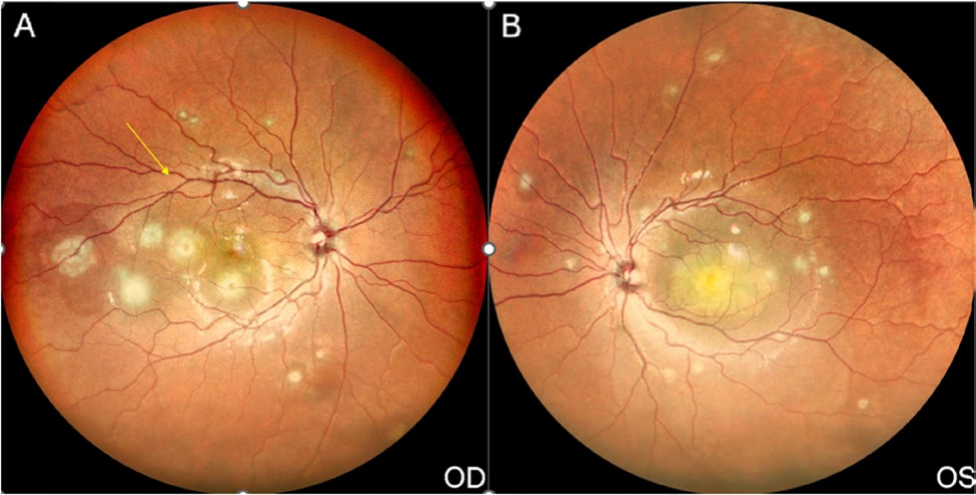
**A **Right eye fundus photo showing multiple choroidal granulomas in the macula; **B** Left eye fundus photo showing a large choroidal granuloma in the macula

The best-corrected visual acuity (BCVA) in the right eye (RE) was 6/12, N6, and 1/60, < N36 in the left eye (LE). Anterior segment examination showed conjunctival congestion in the RE. The rest of the anterior segment examination was unremarkable. The intraocular pressure was 14 mm Hg in the RE and 10 mm Hg in the LE (Goldmann applanation tonometer). Fundus examination of the RE showed a healthy optic disc with multiple subretinal yellowish lesions with ill-defined margins, with central clearing varying in size from half-disc to two-disc diameters (Fig. 2A). The LE fundus showed a subretinal elevated yellowish lesion with ill-defined margins and central clearing at the macula involving the fovea, measuring around ten disc-diameters, with multiple small subretinal yellowish lesions spread throughout the posterior pole and until the mid-periphery (Fig. 2B).

**Fig. 3:**
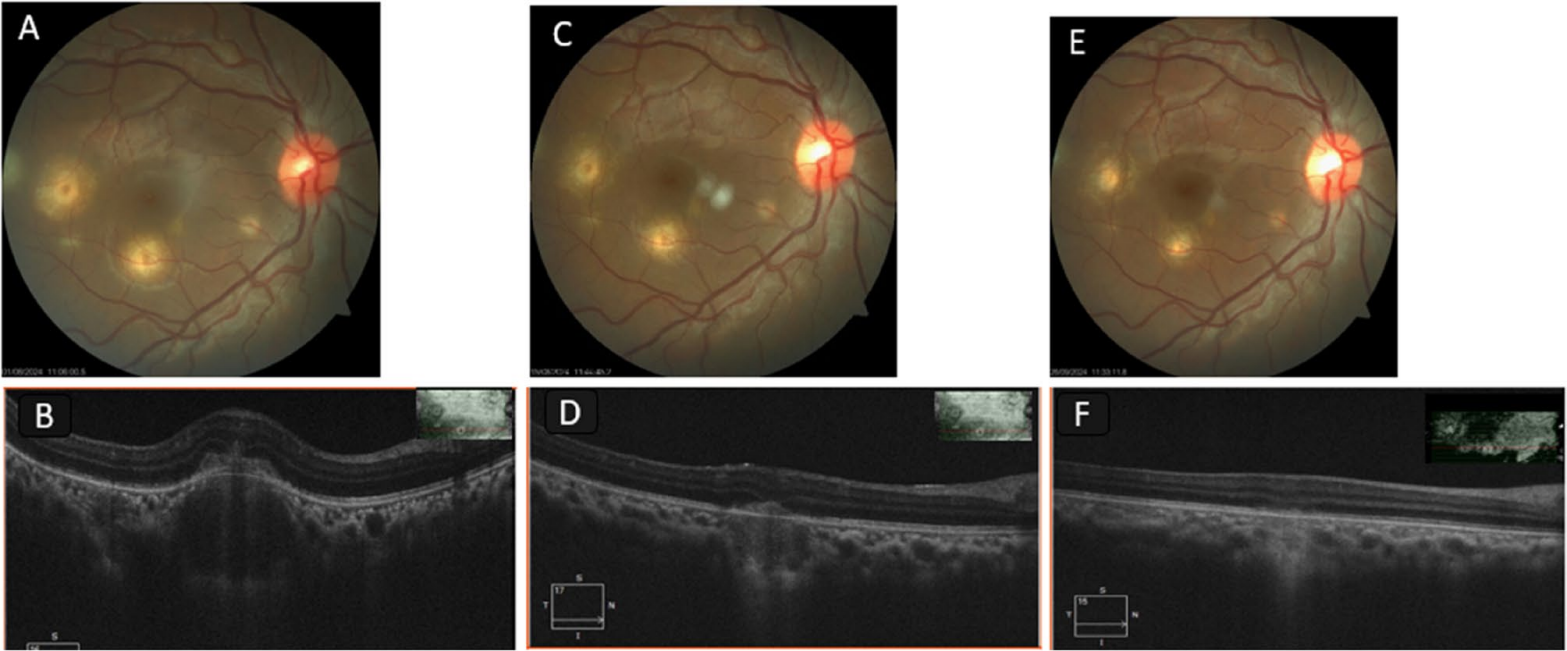
**A **Right eye fundus photo showing multiple choroidal granulomas (**B**) OCT scan through the tubercle along the inferior arcade showingchoroidal bump and subretinal fluid. **C** Fundus photo showing regression of the granuloma on 4th week follow up (**D**) OCT scan through the lesion showing decrease in choroidal bump and with subretinal fluid. **E** Fundus photo showing completely regressed granuloma on 2nd month follow up (**F**) OCT scan through the granuloma showing regression of choroidal bump

**Fig. 4:**
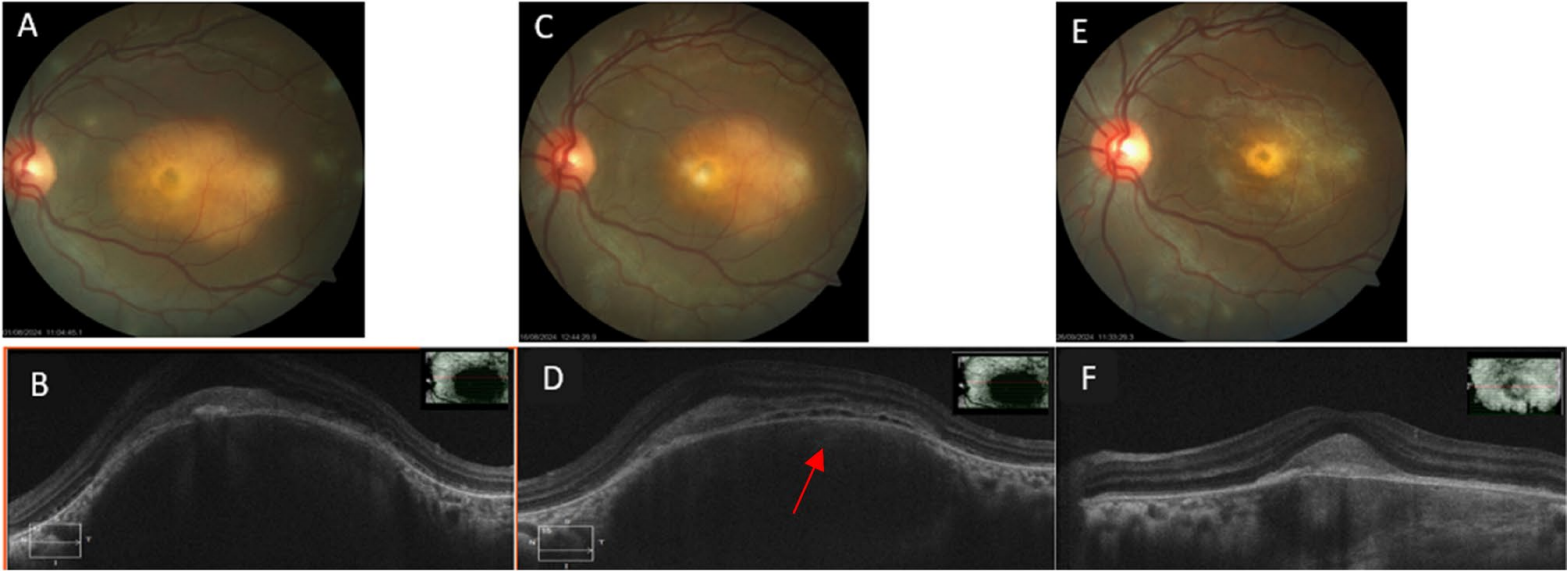
**A **Left eye fundus photo showing a large choroidal granuloma 5*3 mm (**B**) OCT macular scan showing choroidal bump and subretinal fluid (red arrow). **C** Fundus photo showing regression of the granuloma on 4th week follow-up (D) OCT macular scan showing choroidal bump with intraretinal cystic spaces and with subretinal fluid. **E** Fundus photo showing completely regressed granuloma on 2nd month follow up (**F**) OCT scan through the granuloma showing regression of choroidal bump

Optical coherence tomography (OCT) of the RE showed normal foveal contour and elevation of retinal layers with an underlying choroidal bump with hyperreflectivity of the outer retinal layers at the lesion site (Fig. 3B). OCT of the LE through the lesions revealed similar findings, with elevation of retinal layers and underlying dome-shaped choroidal bump with hyperreflectivity of outer retinal layers at the macula (Fig. 4B).

HRCT chest showed bilateral diffuse multiple tiny nodular lesions in the lung parenchyma, suggestive of miliary TB (Fig. 2). Human Immunodeficiency Virus (HIV) and Hepatitis B surface antigen (HbsAg) were nonreactive, and the rest of the uveitis workup was negative. Ultrasonography of the abdomen showed splenomegaly. The systemic and ocular investigations were suggestive of ocular tuberculosis (TB) in the form of bilateral choroidal granuloma and disseminated tubercles. Since the granuloma in the LE was significantly larger and involved the macula, we decided to inject the left eye with intravitreal anti-VEGF and moxifloxacin to facilitate early resolution. The granulomas of the RE were relatively small and discrete, and we decided to monitor their resolution with the continuation of the intensive phase of ATT and oral steroids (1 mg/Kg body weight).

After informed consent, she underwent intravitreal injection of the anti-VEGF drug bevacizumab (1.25 mg/0.05 mL) (off-label use) with moxifloxacin (500 µg/0.1 mL) (off-label use) in the LE under topical anaesthesia. One week after injection, the BCVA of the left eye remained the same at 1/60. However, the lesion appeared to be consolidating. She was continued on weekly intravitreal injections. At 3-week follow-up, her BCVA in the RE improved to 6/9 N6, and her BCVA in the LE improved to 2/60 < N36. The OCT through the granuloma in the LE showed a decrease in the choroidal bump and a reduction of subretinal fluid, suggesting consolidation of the lesion (Fig. 4D). The lesions in the RE also appeared to be getting well-defined and healing (Fig. 3C). The oral steroids were tapered, and the patient was continued on the intensive phase of ATT. However, on closer examination of the OCT of the LE, there were a few intraretinal cysts (Fig. 4D) at the base of the granuloma, suggestive of continued activity, so it was decided to continue with the weekly intravitreal injections. After seven weekly intravitreal injections, her BCVA improved to 6/36, < N36 in the LE, and the choroidal bump decreased in size (Fig. 4F). The intensive phase of ATT was stopped after 2 months, and the patient was shifted to the continuation phase for 9 months. The oral steroids were tapered in a weekly fashion and stopped after about 10 weeks of treatment.

On the last follow-up, 5 months after starting treatment, her systemic condition improved, with the healing of her pulmonary lesions. The ocular lesions appeared scarred, and the patient maintained a vision of RE 6/6, N6 and LE 6/24-, N18. The patient continued her continuation phase of ATT and is on two monthly follow-ups.

### Discussion and conclusion

Choroidal tuberculomas are usually large, unilateral, solitary, yellowish lesions at the posterior pole.Tuberculomas have predilection for the foveal and perifoveal area. They may vary in their presentation with features such as associated haemorrhage, striae, or exudative retinal detachment. Choroidal tubercles are generally small and multiple and have less defined borders than granulomas, with more surrounding edema. They have an increased chance of association with central nervous system (CNS) TB [4]. Mehta and associates found choroidal tubercles in 34.6% of 52 patients with neurotuberculosis [5]. 

Pathologically speaking, granulomas are chronic collections of inflammatory cells created by the immune system to prevent the dissemination of Mycobacterium tuberculosis.

Immune responses skewed towards T helper 2 (Th2) cells cross-inhibit protective responses such as granuloma formation, which may have a critical role in miliary tuberculosis [6]. In a study by Mehta et al. on the patterns of ocular inflammation in patients with miliary tuberculosis(TB), all 22 eyes of the patients with miliary TB included showed no signs of inflammation on slit lamp examination and single or multiple choroidal tubercles bilaterally in 7 patients and unilaterally in 4 patients with no associated vitritis or raised intraocular pressure [7]. 

In large TB granuloma, local ischemia leads to upregulation in vascular endothelial growth factor (VEGF) production at retinal pigment epithelium (RPE) and photoreceptor levels [3]. Due to increased VEGF production, TB granulomas are associated with high vascularity and angiogenesis [2]. 

Hence, for the effective treatment of these granulomas, specifically, larger granulomas, macula-involving or threatening granulomas, we must treat the infection and the inflammation. Combining this treatment along with ATT and systemic steroids is recommended, as intravitreal treatment aids in the direct attack on the inflammation and infection, hastening recovery [3]. 

Due to increased vascular endothelial growth factor (VEGF) production, TB granulomas are associated with high vascularity and angiogenesis [1]. However, tuberculomas resist both ATT and oral steroid combination due to abnormal vascularisation, maintaining the inflammatory process. Production of VEGF occurs as a part of inflammation to promote vessel dilation and in response to hypoxia. VEGF is a known biomarker for active disease in pulmonary and extrapulmonary sites [8]. Anti-VEGF therapy has been used to treat TB granulomas, which are highly vascular, associated with exudation or massive serous retinal detachment, and refractory to conventional ATT and corticosteroids [8]. Parallel reduction of VEGF with clinical regression of the granuloma has been reported [7]. There was a significant correlation between decreasing levels of aqueous VEGF-A and the clinical regression of these tubercular granulomas [9]. Moxifloxacin belongs to the fluoroquinolone group, which consists of second-line ATT drugs [9]. Intravitreal moxifloxacin has been used in the management of endophthalmitis. Recent studies using high-performance liquid chromatography coupled with tandem mass spectrometry (HPLC-MS/MS), applied in rabbit models of tuberculosis, have revealed that moxifloxacin exhibits markedly enhanced intracellular accumulation within macrophages, characterized by a substantially elevated intracellular-to-extracellular concentration ratio relative to first-line anti-tubercular agents such as isoniazid, rifampicin, and pyrazinamide [10]. However, moxifloxacin demonstrates limited diffusion into the necrotic core of the granuloma, which is effectively penetrated only by pyrazinamide. Consequently, we employed intravitreal administration of moxifloxacin to specifically target the metabolically active component of the tubercular granuloma, namely the activated macrophages harbouring *Mycobacterium tuberculosis*, thereby facilitating rapid microbial clearance. High concentrations of VEGF in these patients warrant frequent administration of intravitreal anti-VEGF. The half-life of bevacizumab has a mean of 4.9 days [11]. These factors suggest a weekly dose of anti-VEGF and moxifloxacin to control the disease process. In a study by Agarwal et al. [12], weekly intravitreal injections of anti-VEGF bevacizumab with moxifloxacin in addition to the standard treatment with oral steroids and ATT caused prompt resolution of tubercular granulomas in a series of 10 patients with a mean number of injections of 3.1.

In conclusion, we report a rare manifestation of a case of miliary tuberculosis with bilateral multiple TB granulomas. We highlight the prompt response in both eyes with ATT and oral steroids and emphasize the adjuvant role of weekly intravitreal anti-VEGF and moxifloxacin in achieving faster resolution in these challenging patients.

## Data Availability

No datasets were generated or analysed during the current study.

## Abbreviations

ATT: Anti Tubercular Treatment
TB: Tuberculosis
HRCT: High-Resolution Computed Tomography
VEGF: Vascular Endothelial Growth Factor
BCVA: Best Corrected Visual Acuity
